# Arterial enhancement fraction in evaluating the therapeutic effect and survival for hepatocellular carcinoma patients treated with DEB-TACE

**DOI:** 10.1186/s40644-022-00477-z

**Published:** 2022-07-30

**Authors:** Bin Chai, Dongqiao Xiang, Wei Wang, Yanqiao Ren, Fuquan Wang, Jihua Wang, Guofeng Zhou, Chuansheng Zheng

**Affiliations:** 1 Department of Radiology, Union Hospital, Tongji Medical College, Huazhong University of Science and Technology, Wuhan, China; 2 Hubei Province Key Laboratory of Molecular Imaging, Wuhan, China

**Keywords:** Hepatocellular carcinoma, Transarterial chemoembolization, Computed tomography, Quantitative evaluation, Survival analysis

## Abstract

**Background:**

Arterial enhancement fraction (AEF), derived from triphasic CT scans, is considered to indirectly reflect the ratio of hepatic arterial perfusion to total perfusion. The purpose of this study was to retrospectively investigate the relationship between AEF and treatment response and survival in hepatocellular carcinoma (HCC) patients treated with drug-eluting bead (DEB) TACE.

**Methods:**

AEF of primary lesion (AEF_pre_) and residual tumor (AEF_post_) in 158 HCC patients were obtained from triphasic liver CT examinations pre- and post-treatment. Wilcoxon-signed rank test was used to compare the AEF_pre_ and AEF_post_ for different response groups. Survival curves for overall survival (OS) in patients with different AEF were created by using Kaplan-Meier method. Cox regression analyses were used to determine the association between AEF and OS.

**Results:**

There was no correlation between AEF_pre_ and treatment response. After DEB-TACE, AEF_post_ was significantly lower than AEF_pre_ either in the partial response group (38.9% vs. 52.7%, *p* <  0.001) or in the stable disease group (49.3% vs. 52.1%, *p* = 0.029). In the progression disease group, AEF_post_ was numerically higher than AEF_pre_ (55.5% vs. 53.0%, *p* = 0.604). Cox regression analyses showed that risk of death increased in patients with AEF_pre_ > 57.95% (HR = 1.66, *p* = 0.019) or AEF_post_ > 54.85% (HR = 2.47, *p* <  0.001), and the risk reduced in patients with any reduction in tumor AEF (decrease ratio *≥* 0) and with increased AEF but not exceeding the ratio of 0.102 (increase ratio <  0.102) (HR = 0.32, *p* <  0.001).

**Conclusions:**

The change in AEF of viable tumor is correlated with response of HCC to DEB-TACE. In addition, the AEF could be a helpful predictor in future studies on the embolization treatment for HCC.

## Background

Hepatocellular carcinoma (HCC) is the third most common cause of cancer-related death worldwide [1]. Catheter-based locoregional treatment, also known as transarterial chemoembolization (TACE), is the most frequently implemented in patients with unresectable HCC across all disease stages [2]. The modified response evaluation criteria in solid tumors (mRECIST) was developed to assess response to TACE by measuring the shrinkage of viable tumor, seen as a decrease in contrast-enhancing areas at conventional contrast-enhanced imaging [3]. However, the size-based criteria do not take the quantity of enhancement into account and show impaired capability in some non-measurable HCC lesions [4, 5].

Computed tomography perfusion imaging (CTPI) provides quantitative information about the hemodynamics properties of tissue. The potential value of CTPI in response evaluation and prognostic prediction for HCC treated with TACE has been investigated by several studies [6–9]. Among the various perfusion values that can be calculated with CTPI, hepatic perfusion index (HPI), the ratio of hepatic arterial perfusion to total perfusion, is regarded as an essential parameter for characterizing the hemodynamic features of HCC [10]. Liver CTPI typically involves scanning the liver at numerous (> 20) time points after IV contrast injection, thus requiring dedicated scanning protocols and a large radiation dose [11].

Arterial enhancement fraction (AEF) derived from routine triphasic liver CT examinations, introduced by Kim et al. [12], is an ideal surrogate biomarker for HPI and therefore addresses the issue of radiation dose. They defined AEF as the ratio of the absolute increment of attenuation in the arterial phase to that of the portal venous phase: AEF = [(HU_A_ - HU_U_)/(HU_P_ - HU_U_)] × 100%, where HU, A, P, and U stood for attenuation, arterial phase, portal phase, and unenhanced, respectively. Using the quantitative color mapping of AEF, they increased the sensitivity for HCC detection from 71.7 to 88.8% [12]. The strong correlation (*r* = 0.91, *p* <  0.001) between HPI and AEF was then observed in 10 rabbits with VX2 liver tumor by the same team [10]. Several authors have discussed the possibility of using AEF to assess the efficacy of chemotherapy, radiofrequency ablation, and radioembolization in liver metastases disease [13–15]. Thus, we hypothesize that AEF would also be a feasible biomarker to evaluate the devascularization effect induced by TACE.

The current study aims to assess changes in AEF of viable tumor after drug-eluting bead (DEB) TACE. Furthermore, we seek to investigate the relationships between AEF and response and survival of HCC patients treated with DEB-TACE.

## Methods

### Patient selection

**Fig. 1:**
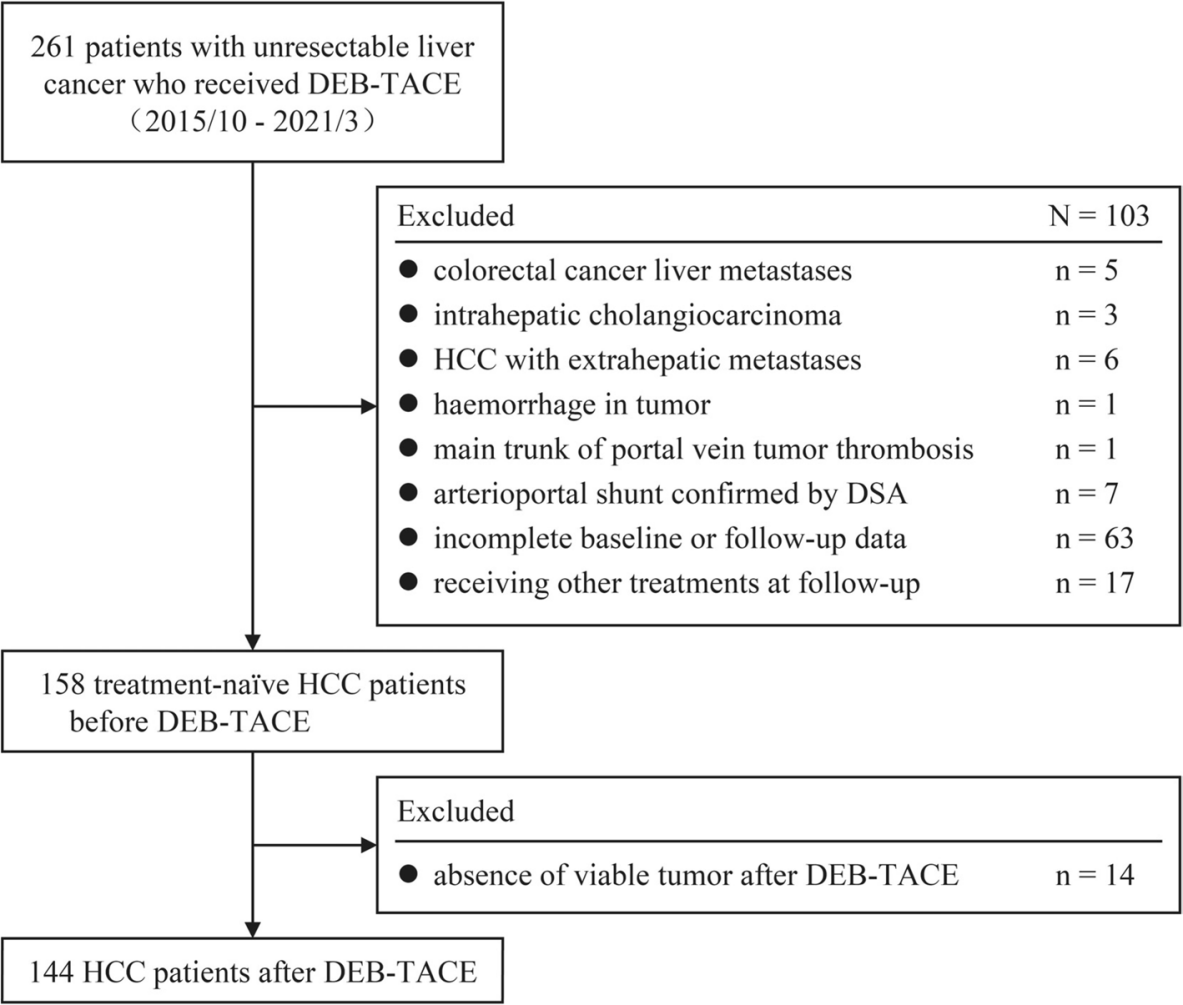
Flowchart showing the patient selection. HCC lesions with haemorrhage, main trunk tumor thrombosis of portal vein, or arterioportal shunt were not eligible for creating quantitative color mapping and therefore excluded. Fourteen patients who obtained complete response were excluded from post-treatment analyses because of the absence of residual tumors. DEB-TACE: drug-eluting bead transarterial chemoembolization; HCC: hepatocellular carcinoma; DSA: digital subtraction angiography

Between October 2015 and March 2021, 261 consecutive treatment-naïve patients with unresectable liver cancer who received DEB-TACE were identified from the electronic medical database of Wuhan Union Hospital. Of the 261 patients, 103 were excluded owing to: (a) histology other than HCC (*n* = 8) or (b) extrahepatic metastases (*n* = 6) or (c) conditions not eligible for measuring AEF, including haemorrhage in tumor (*n* = 1), main trunk of portal vein tumor thrombosis (*n* = 1), arterioportal shunt (*n* = 7) or (d) absence of triphasic CT scan data at baseline or at follow-up (*n* = 63), or (e) receiving any treatments other than TACE at follow-up (*n* = 17). The diagnosis of HCC was either biopsy-proven or met the European Association for the Study of the Liver (EASL) imaging criteria [16]. Eventually, 158 treatment-naïve HCC patients treated with DEB-TACE were enrolled for pre-treatment analyses. Of note, fourteen patients who obtained complete response (CR) after initial DEB-TACE were further excluded for the absence of viable residual tumor, leaving 144 patients for post-treatment analysis (Fig. 1). Five patients who previously underwent partial hepatectomy for HCC were confirmed without recurrence during at least 2 years of follow-up, and therefore HCC lesions of theirs were regarded as de-novo tumors [17].

### DEB-TACE technique and follow-up protocol

All DEB-TACE procedures were performed by a team of interventional radiologists with no less than 10 years of experience. For the treatment, CalliSpheres (Jiangsu Hengrui Medicine Co. Ltd., Jiangsu, China) beads of two different sizes (100–300 μm or 300–500 μm) were loaded with 60 or 80 mg of epirubicin per vial (1 g DEB) and mixed with non-ionic contrast medium to obtain the final injectable beads. After local anesthesia, transfemoral access was gained, and a 5-F visceral catheter (Yashiro, TERUMO, Japan; or R-H, COOK, USA) was advanced into the coeliac axis to identify the arterial blood supply. Superior mesenteric arterial portovenography was also performed to confirm the patency of the portal vein. Then, a coaxial 2.7-F microcatheter (Progreat, Terumo, Japan) was superselectively placed into the feeding arteries of tumors for embolization in all patients. The DEB were administrated up to a maximum of two vials, and further embolization was performed with non-resorbable bland microparticles if needed. Finally, angiography was performed to determine whether vascular stasis was achieved.

The pre-treatment examinations included liver function and alpha-fetoprotein (AFP). The baseline CT scan of the liver was scheduled within 2 weeks before treatment. Patients were followed up with triphasic CT an average of 46 days after initial treatment, and “on-demand” TACE procedures (DEB or conventional) were scheduled at an interval of 6 to 12 weeks upon the demonstration of viable tumors or intrahepatic recurrences by CT unless there was evidence of contraindications. Antiangiogenesis therapy was recommended once radiological progression (according to mRECIST) occurred (Sorafenib as initial treatment and Apatinib if the former failed) unless there was evidence of contraindications. The last follow-up date was September 30, 2021.

### Image and AEF acquisition

**Fig. 2:**
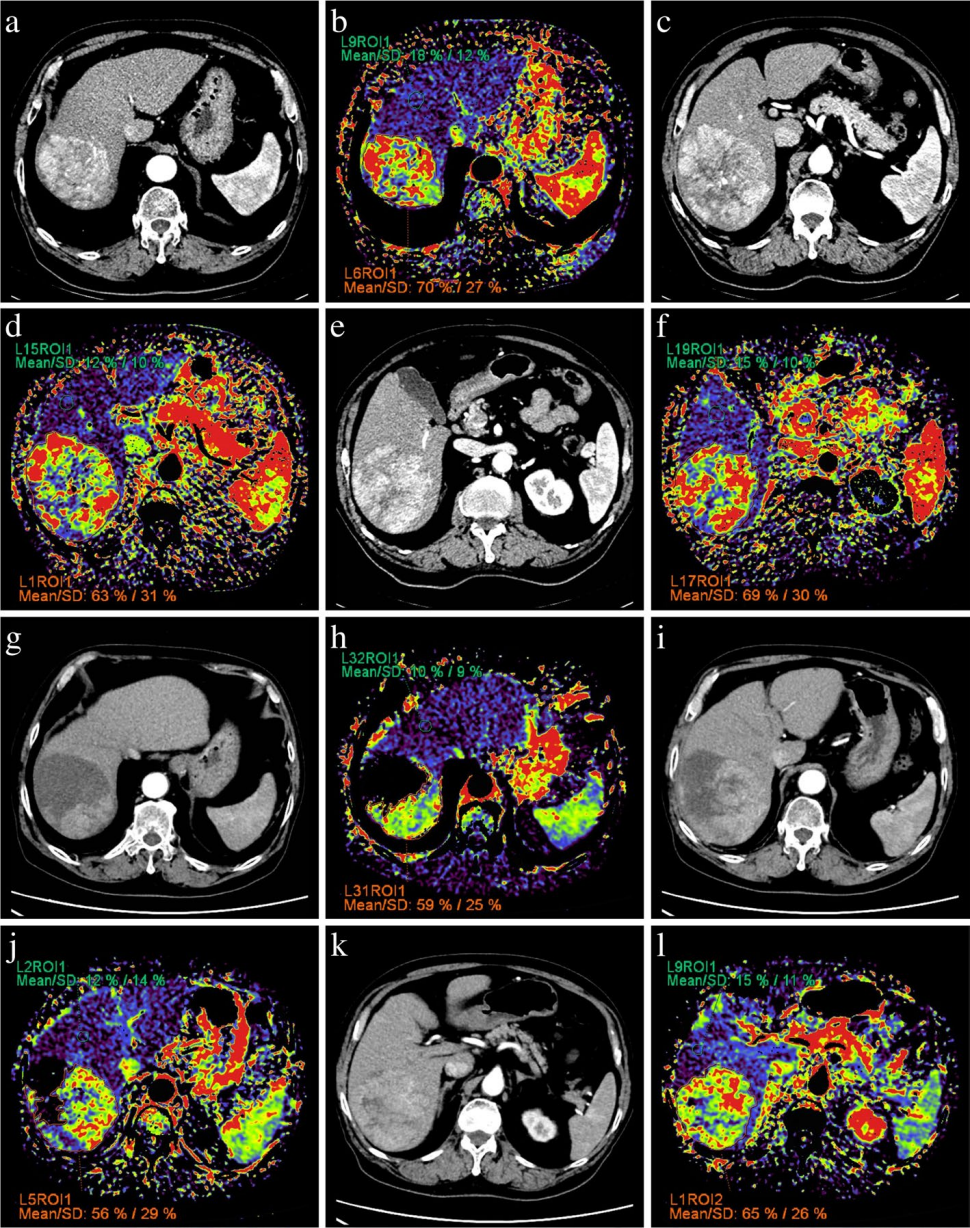
Arterial phase images and corresponding AEF color maps of three representative transverse planes 4 days before (**a**-**f**) and 34 days after ( **g**- **l**) DEB-TACE from a 76 years old, male, HCC patient who had SD response according to mRECIST. Before treatment, a heterogeneously enhanced HCC lesion was located at the right lobe, with significantly higher AEF ($\frac{70\%+63\%+69\%}{3}=67.3\%$, showed as red and yellow region) than surrounding liver parenchyma ($\frac{18\%+12\%+15\%}{3}=15.0\%$, showed as blue and purple region). After treatment, the necrosis of tumor induce by embolization showed as signal loss on the AEF color map, whereas the AEF of residual viable tumor ($\frac{59\%+56\%+65\%}{3}=60.0\%$) remain higher than surrounding parenchyma ($\frac{10\%+12\%+15\%}{3}=12.0\%$). DEB-TACE: drug-eluting bead transarterial chemoembolization; HCC: hepatocellular carcinoma; SD: stable disease; mRECIST: modified response evaluation criteria in solid tumors; AEF: arterial enhancement fraction

All CT acquisitions were performed on a Somatom Definition AS, a Somatom Definition, or a Somatom Force CT scanner (Siemens Healthcare, Erlangen, Germany). After unenhanced scanning, a triphasic contrast-enhanced scan was performed after intravenous administration of 80–100 mL non-ionic contrast medium (Iopamidol, 370 mg I/mL, Bracco) using power injection at a rate of 2.5–3.0 mL/s followed by saline flush (20 mL). Arterial phase, portal venous phase, and equilibrium phase images were obtained at 30 seconds, 50 seconds, and 3 minutes, respectively. Tube voltage was set at 120 kV with automated tube current modulation. Axial slices of 1.5 mm thick were reconstructed, and a medium smooth convolution kernel (B30f) was used. After image acquisition, the unenhanced, arterial phase, and portal venous phase data sets were transferred to a syngo.via workstation by Siemens Healthcare (Erlangen, Germany). Quantitative color mapping of AEF was then generated using the dedicated AEF tool contained in the MM Oncology mode on the workstation (Fig. 2).

### Image analysis

The primary lesion was determined as the largest measurable target tumor of each patient in the consensus of two radiologists (B.C and D.Q.X) for AEF and mRECIST evaluation. Radiological features of the primary lesion, including diameter, margin (smooth or non-smooth), and macrovascular invasion (presence of portal vein or hepatic vein tumor thrombosis), were reviewed for subgroup analysis. Lesion diameter was estimated by measuring the maximum diameter of viable tumor on the arterial-phase images. Treatment response was classified into CR, partial response (PR), stable disease (SD), and progressive disease (PD) in accord with mRECIST [3].

The AEF of primary HCC lesion pre-treatment (AEF_pre_) and that of residual viable tumor post-treatment (AEF_post_) were obtained from baseline and the first follow-up AEF color map for each patient. After initial DEB-TACE, viable residual tumors were first identified on arterial phase imaging of contrast-enhanced CT and then confirmed in the following TACE procedure. The decrease ratio of AEF was defined as (AEF_pre_ – AEF_post_)/AEF_pre_. The region of interest (ROI) of the viable tumor was manually drawn in three representative transverse planes, and the mean AEF of three sections was used for further analysis. On each representative plane, an ROI of 1 × 1 cm^2^ was placed 4-5 cm away from the tumor without containing major vessels to calculate the mean of tumor-free liver parenchyma. The consensus on ROI drawing was achieved by two radiologists mentioned above who had participated in the lesion confirmation.

### Statistical analysis

After testing for normality using the Kolmogorov-Smirnov test, AEF values were expressed by mean ± standard deviation (SD). Categorical variables were expressed as frequencies (percentage). Student’s t-tests were used to compare AEF_pre_ or AEF_post_ between clinical and radiological subgroups. We compared the AEF of the different response groups using analyses of variance (ANOVA), and the Holm-Bonferroni correction was performed for the post hoc test [18]. The Wilcoxon-signed rank test was used to assess the differences between AEF_pre_ and AEF_post_. The Pearson’s product-moment correlation coefficients (r) were determined for the magnitude of the relationship between AEF_pre_ and lesion diameter, and the Spearman rank correlation coefficients (ρ) for that between AEF_post_ or decrease ratio and treatment response. Overall survival (OS) was defined as the interval between the first DEB-TACE procedure and death or the last follow-up (considered censored). The best cutoff values for AEF_pre_, AEF_post_, and decrease ratio were determined by *Cutoff Finder*, a web application (http://molpath.charite.de/cutoff) developed by Budczies J et al. [19], to identify the patients with favorable and unfavorable survival outcomes. The cutoff optimization was based on the point with the most significant (log-rank test) split. Survival curves for OS were created according to the Kaplan-Meier method. Uni- and multivariate Cox regression analyses were performed to estimate the influence of AEF and possible confounding factors on OS, including age, gender, Child-Pugh class, baseline AFP levels, lesion number, lesion diameter, macrovascular invasion, treatment response, and repeated TACE treatment courses after the initial DEB-TACE. A *p*-value of less than 0.05 was considered significant. R software version 4.1.2 (http://cran.r-project.org) was used for all statistical analyses.

## Results

Demographics, underlying liver disease, and information about tumor-related details of the 158 patients are depicted in Table 1. Hepatitis B virus infection was the most common cause of HCC (*n* = 136, 86.1%), other causes included hepatitis C (*n* = 2), liver flukes infection (*n* = 3), cirrhosis caused by chronic Budd-Chiari syndrome (*n* = 1), and other unknown causes (*n* = 16). The degree of the macrovascular invasion incorporated Vp1 or Vp2 portal vein tumor thrombosis (PVTT) according to the Japanese Society of Hepatology PVTT classification [20] (*n* = 56) and hepatic vein tumor thrombosis (*n* = 3).

**Table 1:** Baseline characteristics and treatment response of 158 patients with HCC

Characteristics	No. of patients
Median age (y)	56 (24, 84)^a^
Gender	
Male	137 (86.7%)
Female	21 (13.3%)
Child-Pugh class	
A	123 (77.8%)
B	35 (22.2%)
Cause of HCC	
Hepatitis B	136 (86.1%)
Other	22 (13.9%)
AFP (ng/ml)	
≤ 400	91 (57.6%)
> 400	67 (42.4%)
No. of lesions	
Solitary	76 (48.1%)
Multifocal	82 (51.9%)
Diameter (cm)	9.2 ± 4.2
Macrovascular invasion	
Absent	99 (62.7%)
Present	59 (37.3%)
Treatment response	
CR	14 (8.8%)
PR	53 (33.5%)
SD	58 (36.7%)
PD	33 (21.0%)

Unless otherwise indicated, data in parentheses are percentages

*HCC* Hepatocellular carcinoma, *AFP* α-fetoprotein, *CR* Complete response, *PR* Partial response, *SD* Stable disease, *PD* Progressive disease

^a^Data in parentheses are range

### Quantitative color mapping of the AEF of HCC

#### Before DEB-TACE

Before treatment, the Wilcoxon-signed rank test showed the mean AEF_pre_ was significantly higher than AEF of tumor-free parenchyma (52.6% ± 14.2% vs. 19.3% ± 7.5%, *p* <  0.001). Only AEF_pre_ in patients with Child class B was significantly higher than AEF_pre_ in those with Child class A (*p* = 0.012), while no statistically significant differences in AEF_pre_ were observed between any other subgroups (Table 2). The AEF of tumor-free parenchyma in patients with Child class A was slightly lower than in those with Child class B (19.1% ± 7.9% vs. 20.0% ± 5.4%, *p* = 0.498). According to the result of ANOVA, there was no correlation between AEF_pre_ and treatment response (*p* = 0.988) (Table 2). The Pearson correlation test showed no linear correlation between AEF_pre_ and tumor diameter (*r* = 0.05, *p* = 0.551).

**Table 2:** AEF of viable tumor before and after DEB-TACE

	Whole patients (*n* = 158)		Patients excluding CR response (*n* = 144)			
	AEF_pre_ (%)	p*	AEF_pre_ (%)	AEF_post_ (%)	Decrease ratio	*p*†
Whole patients	52.6 ± 14.2	–	52.5 ± 14.2	46.9 ± 16.5	0.12 (− 0.02, 0.26)	**< 0.001**
Child-Pugh class						
A	51.1 ± 13.9	**0.012**	51.1 ± 14.1	46.3 ± 15.8	0.09 (− 0.05, 0.26)	**< 0.001**
B	57.9 ± 14.1		58.0 ± 13.4	49.2 ± 19.1	0.14 (0.04, 0.30)	**0.002**
AFP (ng/ml)						
≤ 400	51.4 ± 14.9	0.279	51.4 ± 15.1	44.1 ± 16.9	0.14 (0.04, 0.26)	**< 0.001**
> 400	54.2 ± 13.1		54.0 ± 13.1	50.3 ± 15.6	0.06 (− 0.12, 0.27)	**0.019**
Lesion margin						
Smooth	53.8 ± 12.9	0.219	53.9 ± 12.9	45.1 ± 16.5	0.17 (0.04, 0.29)	**< 0.001**
Non-smooth	51.0 ± 15.6		51.0 ± 15.5	49.0 ± 16.5	0.06 (− 0.13, 0.22)	0.143
Macrovascular invasion invasion						
Absent	52.9 ± 14.3	0.758	52.8 ± 14.3	43.6 ± 16.8	0.17 (0.05, 0.29)	**< 0.001**
Present	52.2 ± 14.1		52.2 ± 14.2	51.8 ± 14.9	0.05 (−0.20, 0.17)	0.516
Treatment response						
CR	53.3 ± 14.6	0.988	–	–	–	–
PR	52.7 ± 13.3		52.7 ± 13.3	38.9 ± 13.8	0.26 (0.18, 0.37)	**< 0.001**
SD	52.1 ± 15.0		52.1 ± 15.0	49.3 ± 16.1	0.05 (−0.03, 0.11)	**0.029**
PD	53.0 ± 14.6		53.0 ± 14.6	55.5 ± 16.0	0.07 (−0.31, 0.17)	0.604

Decrease ratio was expressed by median (interquartile range) because the distribution of it observed skewed

*AEF* Arterial enhancement fraction, *AFP* α-fetoprotein, *CR* Complete response, *PR* Partial response, *SD* Stable disease, *PD* Progressive disease

* *p* value for differences of AEF_pre_ between subgroups

† *p* value for differences between AEF_pre_ and AEF_post_ in patients without CR response

#### After DEB-TACE

**Fig. 3:**
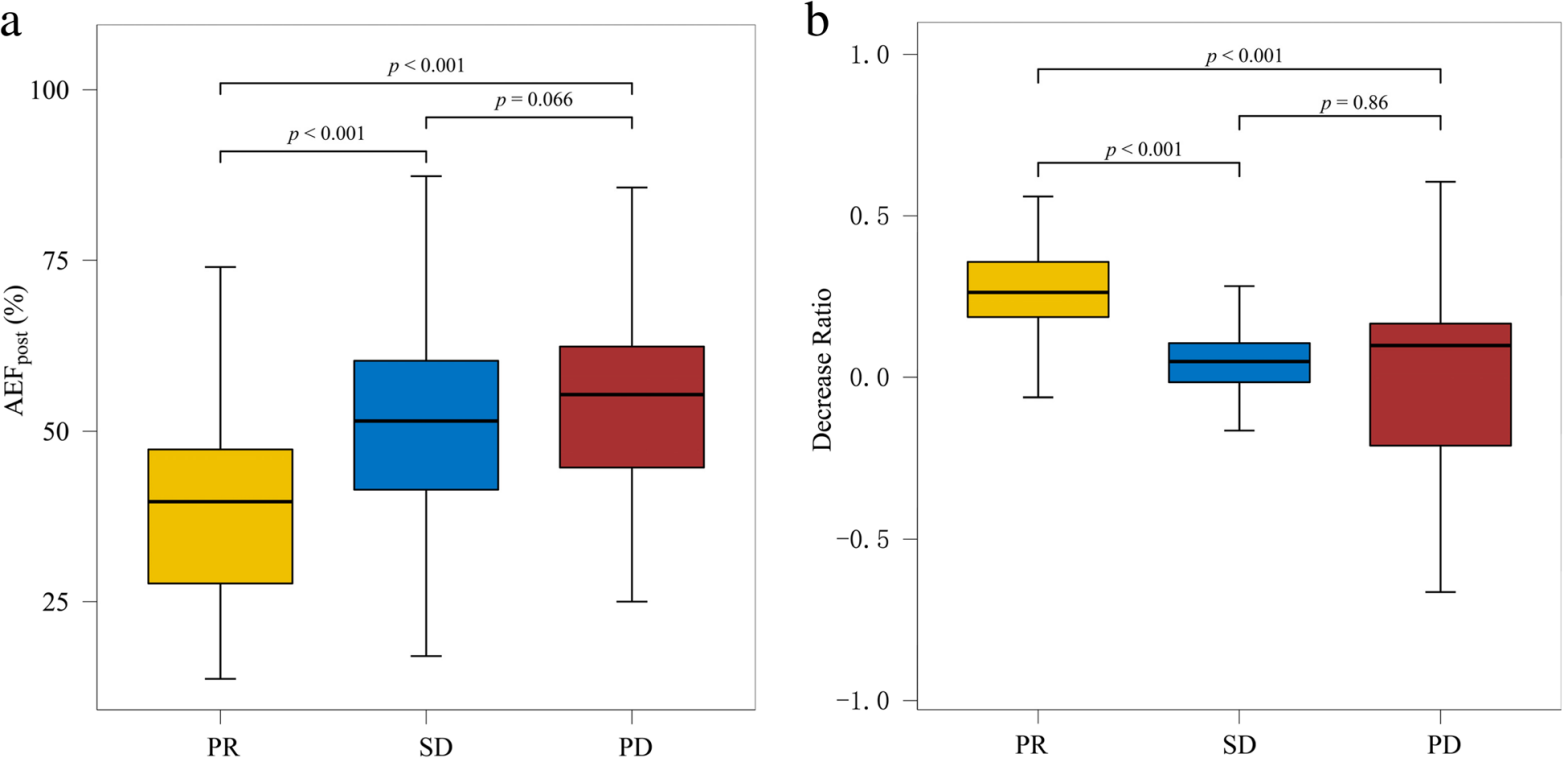
Box plots of the differences in AEF_post_ (**a**) and decrease ratio (**b**) for response groups. The difference in AEF_post_ across response groups was statistically significant, demonstrated by ANOVA (*p* <  0.001), and the statistically significant difference was also observed in decrease ratio across response groups demonstrated by Kruskal-Wallis rank sum test (*p* <  0.001). *P* values for multiple comparisons were corrected by Holm-Bonferroni method. AEF = arterial enhancement fraction; PR = partial response; SD = stable disease; PD = progressive disease; ANOVA: analyses of variance

Fourteen patients obtained CR response after initial treatment, and their triphasic CT imaging of post-treatment lesion displayed a complete absence of enhancement. Lesions on their post-treatment color mappings displayed as signal loss areas so that neither AEF_post_ nor decrease ratio of AEF could be determined. Of 144 patients excluding CR response, AEF_post_ was significantly lower than AEF_pre_ (46.9% ± 16.5% vs. 52.5% ± 14.2%, *p* <  0.001) (Table 2). However, no statistically significant differences between AEF_post_ and AEF_pre_ were observed in patients with non-smooth lesion margin (49.0% ± 16.5% vs. 51.0% ± 15.5%, *p* = 0.143) and presence of macrovascular invasion (51.8% ± 14.9% vs. 52.2% ± 14.2%, *p* = 0.516). In the patients with PD response, AEF_post_ was even numerically higher than AEF_pre_ (55.5% ± 16.0% vs. 53.0% ± 14.6%, *p* = 0.604) (Table 2). Although no difference was observed in AEF_pre_ between any two response groups, the AEF_post_ did show a pattern of rising as response worsened, corresponding with the decline of decrease ratio (Fig. 3). Spearman correlation test showed that AEF_post_ was positively correlated with treatment response (*ρ* = 0.39, *p* <  0.001), and decrease ratio was negatively correlated with treatment response (*ρ* = − 0.47, *p* <  0.001).

### Survival analysis

**Fig. 4:**
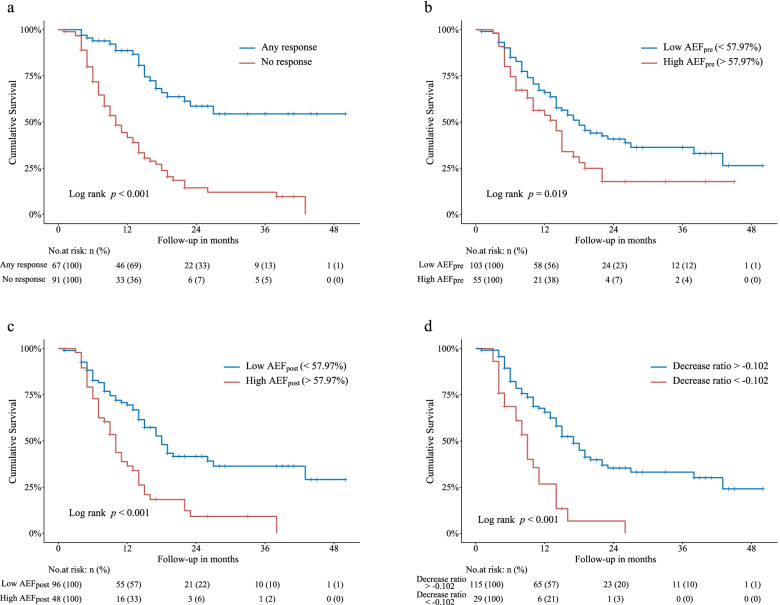
Kaplan-Meier curves showed overall survival of HCC patients stratified by treatment response (**a**), AEF_pre_ (**b**), AEF_post_ (**c**), and decrease ratio (**d**). Analyses in **a** and **b** were based on 158 patients, and analyses in **c** and **d** were based on 144 patients excluding CR response. Decrease ratio > − 0.102 (**d**) contained two parts, decrease ratio ≥ 0 and increase ratio <  0.102. Likewise, decrease ratio < − 0.102 was equivalent to increase ratio > 0.102. HCC: hepatocellular carcinoma; AEF = arterial enhancement fraction; CR: complete response

At the time of data closure on August 31, 2021, 91 patients (57.6%) had died among all 158 patients. The median follow-up duration was 25 months, and the median OS was 15 months. The 6-month, 1-year, and 2-year survival rates were 81, 62, and 33%, respectively. The median of repeated TACE treatment courses patients underwent was two (range, 0–11 courses, not including the initial treatment) during the study period. In the subgroups stratified by treatment response, the OS for any response group (CR and PR) was significantly longer than the no response group (SD and PD) (*p* <  0.001) (Fig. 4a). The optimal cutoff for AEF_pre_, AEF_post_, and decrease ratio, based on the most significant split according to the log-rank test, was determined to be 57.95, 54.85%, − 0.102, respectively (Fig. 4b-d).

Tables 3 and 4 list the results of the univariate and multivariate Cox regression analyses for OS. Univariate analysis in 158 patients showed that risk of death increased in patients with AEF_pre_ > 57.95% (HR = 1.66, *p* = 0.019), and the risk was further increased after confounding factors were introduced for adjustment (HR = 2.29, *p* = 0.001). The higher AEF_post_, namely more than 54.85%, was also shown to elevate the risk of death in the univariate analyses (HR = 2.47, *p* = 0.019) and remained strongly associated with OS in the multivariate analysis (HR = 1.82, *p* = 0.014) (Table 4). On the other hand, reduced risk of death was seen in patients with any reduction in tumor AEF (decrease ratio *≥* 0) and with increased AEF but not exceeding the ratio of 0.102 (increase ratio <  0.102) (HR = 0.32, *p* <  0.001), though the protective effect was marginally significant in the multivariate analysis (HR = 0.61, *p* = 0.068).

**Table 3:** Univariate and multivariate Cox regression analysis for OS in 158 patients

Variables	Univariate		Multivariate	
	HR (95% CI)	*p*	HR (95% CI)	*p*
Age > 55 (years)	0.70 (0.46, 1.06)	0.096	0.95 (0.60, 1.49)	0.818
Male	0.92 (0.52, 1.63)	0.778	1.49 (0.81, 2.76)	0.203
Child–Pugh B class	1.10 (0.65, 1.84)	0.722	1.02 (0.56, 1.83)	0.959
AFP > 400 (ng/ml)	2.24 (1.48, 3.40)	**< 0.001**	1.45 (0.92, 2.28)	0.110
Multifocal disease	2.45 (1.59, 3.77)	**< 0.001**	1.40 (0.87, 2.25)	0.171
Lesion diameter (cm)	1.20 (1.14, 1.26)	**< 0.001**	1.17 (1.10, 1.25)	**< 0.001**
Macrovascular invasion	3.90 (2.54, 5.99)	**< 0.001**	3.45 (2.09, 5.70)	**< 0.001**
Repeated treatment courses	0.79 (0.60, 1.05)	0.099	0.52 (0.37, 0.72)	**< 0.001**
AEF_pre_ > 57.95%	1.66 (1.09, 2.55)	**0.019**	2.29 (1.42, 3.68)	**0.001**

*OS* Overall survival, *HR* Hazard ratio, *AFP* α-fetoprotein, *AEF* Arterial enhancement fraction

**Table 4:** Univariate and multivariate Cox regression analysis for OS in 144 patients excluding CR response

Variables	Univariate		Multivariate^a^		Multivariate^b^	
	HR (95% CI)	*p*	HR (95% CI)	*p*	HR (95% CI)	*p*
Age > 55 (years)	0.67 (0.44, 1.02)	0.063	0.95 (0.60, 1.50)	0.819	0.97 (0.61, 1.53)	0.881
Male	0.94 (0.52, 1.70)	0.843	1.44 (0.76, 2.73)	0.264	1.26 (0.67, 2.39)	0.470
Child–Pugh B class	1.09 (0.64, 1.86)	0.743	1.23 (0.69, 2.17)	0.480	1.43 (0.80, 2.57)	0.232
AFP > 400 (ng/ml)	2.09 (1.37, 3.19)	**0.001**	1.34 (0.84, 2.15)	0.222	1.47 (0.91, 2.37)	0.114
Multifocal disease	2.22 (1.43, 3.45)	**< 0.001**	1.25 (0.74, 2.09)	0.406	1.28 (0.77, 2.14)	0.348
Lesion diameter (cm)	1.18 (1.12, 1.25)	**< 0.001**	1.14 (1.07, 1.22)	**< 0.001**	1.14 (1.07, 1.22)	**< 0.001**
Macrovascular invasion	3.53 (2.28, 5.45)	**< 0.001**	2.79 (1.68, 4.63)	**< 0.001**	2.71 (1.62, 4.53)	**< 0.001**
Repeated treatment courses	0.70 (0.52, 0.93)	**0.013**	0.49 (0.35, 0.70)	**< 0.001**	0.53 (0.38, 0.75)	**< 0.001**
AEF_post_ > 54.85%	2.47 (1.61, 3.78)	**< 0.001**	1.82 (1.13, 2.92)	**0.014**	–	–
Decrease ratio > −0.102	0.32 (0.20, 0.53)	**< 0.001**	–	–	0.61 (0.35, 1.04)	0.068

Decrease ratio > − 0.102 contained two parts, decrease ratio **≥** 0 and increase ratio < 0.102

*OS* Overall survival, *HR* Hazard ratio, *CR* Complete response, *AFP* α-fetoprotein, *AEF* Arterial enhancement fraction

^a^Multivariate analysis for AEF_post_ adjusted by possible confounding factors on OS

^b^Multivariate analysis for decrease ratio adjusted by possible confounding factors on OS

## Discussion

During the evolution of dysplastic nodules to HCC, there was a pattern of hemodynamic change, which involved increasing arterial blood supply due to tumor-related angiogenesis [21]. CTPI enabled the quantification of perfusion characteristics in tumor tissue and has proved a potential technique for assessing the efficacy of various HCC treatments [22–24]. However, the high radiation exposure was one of the most problematic issues limiting the application of this technique, particularly considering that cancer patients may need to undergo repetitive imaging examinations to monitor treatment response [6]. Hepatic AEF, derived from routine triphasic CT scans, was an ideal biomarker that allowed indirect estimation of hepatic arterial perfusion to total perfusion without raising the extra radiation concern. Several studies have demonstrated the application value of AEF on chemotherapy, radiofrequency ablation, and radioembolization in liver metastases disease [13–15].

Our study assessed the changes in AEF of HCC after DEB-TACE and investigated the relationships between AEF and response and survival outcome. The results showed that, before embolization, only AEF of HCC in patients with Child class B was significantly higher than in those with Child class A, while no correlations were observed between AEF and serum marker or tumor characteristics such as diameter. Kaufmann et al. [25] used a CT-based volume perfusion technique to characterize HCC lesions. The result suggested that HPI (a perfusion parameter similar to AEF) was not correlated with lesion size, which was supported by our finding (*r* = 0.05, *p* = 0.551). Kang et al. [26] reported that the AEF of liver parenchyma in Child class A group resembled that in Child class B groups (23.7% ± 7.6% vs. 32.2 ± 10.9%, *p* = 0.16). Though we got a similar result to Kang et al.’s, the difference in AEF between two groups was much less evident in our investigation (19.1% ± 7.9% vs. 20.0% ± 5.4%, *p* = 0.498). It may be attributed to the fact that ROI was delineated based on each liver segment, significantly larger than the ROI area in this study. More importantly, the present study failed to find the association between AEF and treatment response. In other words, no prediction of response was possible before DEB-TACE based on AEF. Nevertheless, Mao et al. [27] succeeded in predicting treatment response by using texture features of HCC on the AEF color map.

The relatively large size of our sample allowed us to divide the patients into different treatment response groups to compare the changes in AEF among them. In the CR group, lesions on their post-treatment color mappings displayed as signal loss areas so that neither AEF_post_ nor decrease ratio of AEF could be determined. In both PR and SD groups, the AEF of the residual tumor was significantly lower than that of the same lesion before embolization (Table 2). However, the decrease ratio of AEF in patients with SD response was significantly lower than in those with PR response (Fig. 3). In the PD group, the AEF of the residual tumor was even numerically higher than that of the original lesion before treatment (Table 2). Correlation analysis also showed that the decrease ratio negatively correlated with treatment response (*ρ* = − 0.47, *p* <  0.001). The trend in AEF across the response groups was similar to previous CTPI research conducted by Chen et al. [24]. They observed, after TACE treatment, the hepatic arterial fraction (HAF, a perfusion parameter used in GE Medical perfusion software, with the exact definition as HPI but calculated with deconvolution model) of HCC lesion significantly decreased in the PR group (63.7% vs. 38.2%, *p* = 0.030), and increased in PD group (45.9% vs. 69.6%, *p* = 0.012), respectively. In the SD group, HAF of residual tumor was numerically lower than HAF before treatment, though the difference did not reach statistical significance (42.3% vs. 33.8%, *p* = 0.248). The possible explanation was that incomplete embolization would cause local hypoxia in the tumor microenvironment, leading to the upregulation of multiple angiogenesis factors and the development of new arterial vessels that sustained tumor relapse. Further histological research was needed to prove such speculation.

Although there was no way to predict treatment response using baseline AEF, we managed to determine cutoff values for AEF_pre_, AEF_post,_ and decrease ratio to identify patients with favorable and unfavorable OS (Fig. 4). The Cox regression analyses suggest that relatively high AEF_pre_ and AEF_post_ result in discouraging survival outcomes. As for the impact of the decrease ratio on the OS, we find that patients would be more likely to gain a significant survival benefit if there is any reduction in tumor AEF after TACE treatment. Surprisingly, the risk remains reduced in patients who obtained an increased AEF with a ratio not exceeding 0.102. However, the impact of the decrease ratio on OS was no longer significant after being adjusted by confounding factors (Table 4).

Other than all of the inherent defects in this research design, our work had some other limitations. Though we speculated that the correlation between AEF and response for DEB-TACE was the consequence of tumor-related angiogenesis, further histological research was needed to prove such speculation. Patients who selected conventional TACE as initial treatment were not included in this study due to the artifact induced by Lipiodol. An MR-based AEF analysis may solve the problem.

## Conclusions

This study revealed that no prediction of response was possible before DEB-TACE based on AEF. After DEB-TACE, the AEF of residual viable tumor and decrease ratio of AEF could be the easily accessible parameters for monitoring the response of HCC to DEB-TACE. In addition, AEF is associated with OS in HCC patients treated with DEB-TACE, which could be a candidate prognostic factor in future studies.

## Data Availability

The datasets used and/or analysed during the current study are available from the corresponding author on reasonable request.

## Abbreviations

HCC: Hepatocellular carcinoma
TACE: Transcatheter chemoembolization
mRECIST: Modified response evaluation criteria in solid tumors
CTPI: Computed tomography perfusion imaging
HPI: Hepatic perfusion index
AEF: Arterial enhancement fraction
DEB: Drug-eluting bead
CR: Complete response
PR: Partial response
SD: Stable disease
PD: Progressive disease
ROI: Region of interest
AFP: α-fetoprotein
ANOVA: Analyses of variance
OS: Overall survival
PVTT: Portal vein tumor thrombosis
HR: Hazard ratio
DSA: Digital subtraction angiography
